# The Impacts of Different Pea Protein Isolate Levels on Functional, Instrumental and Textural Quality Parameters of Duck Meat Batters

**DOI:** 10.3390/foods11111620

**Published:** 2022-05-30

**Authors:** Xueshen Zhu, Beibei Tan, Ke Li, Shaohua Liu, Ying Gu, Tianlan Xia, Yun Bai, Peng Wang, Renlei Wang

**Affiliations:** 1 Key Laboratory of of Biological Functional Molecules of Jiangsu Province, College of Life Science and Chemistry, Jiangsu Second Normal University, Nanjing 211200, China; xueshen.zhu@jssnu.edu.cn (X.Z.); beibeitan624@163.com (B.T.); njulsh@126.com (S.L.); joycemvp2012@163.com (Y.G.); 2 College of Food Science and Technology, Nanjing Agricultural University, Nanjing 210095, China; baiyun@njau.edu.cn; 3 Henan Key Laboratory of Cold Chain Food Quality and Safety Control, College of Food and Bioengineering, Zhengzhou University of Light Industry, Zhengzhou 450001, China; xc_like@163.com; 4 School of Food Science, Nanjing Xiaozhuang University, Nanjing 211171, China; xia_tianlan@126.com

**Keywords:** duck meat batters, pea protein isolate, textural quality, gel-forming ability

## Abstract

This study aimed to investigate the effect of pea protein isolate (PPI) on the functional, instrumental and textural quality parameters of duck meat batters (DMB). Ground duck breast meat was mixed with different concentrations of PPI (0%, 3%, 6% or 9%, *w*/*w*) to prepare DMB. The color, cooking yield, water-holding capacity, water distribution and migration, rheological properties and texture profile of the DMB were evaluated. The results showed that the L* value of the gel decreased and the b* value increased with the increasing pea protein addition. The cooking yield and water-holding capacity showed a gradual increase, but the difference was not significant (*p* > 0.05). Compared with the control, the storage modulus (G′) and loss modulus (G″) were higher at the beginning and at the end and increased with the addition of pea protein, which was in accordance with the Fourier series relationship. The hardness, chewiness and gumminess of the gels gradually increased; on the contrary, the springiness and cohesiveness first increased and then decreased, respectively, reaching a maximum value of 0.96 and 0.81 when the addition amount reached 6%. Adding pea protein to the gels not only increased the area of immobilized water but also decreased the area of free water, thus improving the water-holding capacity of the batters. Therefore, pea protein can promote the formation of a stable and elastic network structure of duck meat batters.

## 1. Introduction

With its high protein and relatively low price, duck meat plays an important role in the meat industry of Asian countries, including Vietnam, Thailand, Indonesia, India and especially China [1]. According to FAO statistics, of the 1.15 billion ducks kept in 2020 worldwide, 89 percent were kept in Asia. The largest duck populations are found in China. Despite the popularity of traditional duck products among both young and old consumers, there is an inherent demand for more diverse duck products [2]. At the same time, people are becoming increasingly concerned about physical health, balanced diets and the impact of husbandry on environment issues; therefore, the partial substitution of plant proteins for animal proteins has become the hottest topic in recent years [3]. Plant meat analogs, edible insects and cultured meat are promising major meat substitutes that could be used as protein sources in the future [4,5], and recently, different textured plant proteins (from peas, sunflowers or pumpkins) have been reported to have different effects on the composition of mince meat products, as well as different environmental effects. The substitution of textured plant proteins for pork proteins may introduce dietary fiber into the meat diet [6]. Among the various non-meat proteins, pea protein is a good choice with low price, high nutritional value, low fat, high protein and low cholesterol, which is more in line with their need to replace traditional animal proteins [7]. Pea proteins are also becoming popular in European countries, as they are currently produced from non-GMOs [8]. Pea protein is known to be highly digestible, hypoallergenic and it contains almost no ingredients that cause intolerance, as it is gluten- and lactose-free. In addition, pea protein has good emulsification and other functional properties [9], and our recent study showed that partial addition improved the properties of duck myofibrillar protein gels and improved their microstructure [10]. The reduction of fat and its replacement with texturized pea protein was feasible, with no detrimental impact on the acceptability of dry, fermented sausages [11] and low-fat frankfurters [12]. However, more research is still needed to expand the utilization of pea protein in duck meat products. Pea proteins have an exemplary hydrophilic oil structure, and during foaming, proteins can adsorb to these interfaces and reduce interfacial tension, and proteins can also form network structures through interactions and impart some stability [13]. Therefore, pea proteins are gaining more and more attention as other meat expanders. To date, various types of functional ingredients, such as starch [14], fiber [15,16] and hydrocolloids [17], are widely used as extenders, fillers or binders in meat products to improve the cooking yield and water retention, optimize the meat texture, bind between meat pieces, and stabilize the water content in pork and broiler emulsions during food preparation and cooking [18]. However, limited literature has been found on the study of the gelation properties of comminuted duck meat products after the addition of PPI.

Therefore, the main objective of this experiment was to investigate the effect of pea protein on the properties of DMB by adding different proportions in order to improve the quality of the gel formed from duck mince and to also broaden the added value of pea protein and expand its application in other areas.

## 2. Materials and Methods

### 2.1. Sample Preparation and Chemicals

Fresh duck breast muscle was obtained from a local farm market in Lishui, Nanjing, China. After the duck breast was stripped from the carcass (carcasses weight, 5.0 ± 0.5 kg), the breast meat (pH, 5.92 ± 0.11) was removed as much as possible, and then, the samples were stored in a refrigerator at −80 °C and sealed in self-sealing bags. Pea protein isolate (80%) was obtained from Yantai Oriental Protein Technology Co (Yantai, China). All chemical reagents were chemically pure (≥99.5%).

Frozen duck breasts (60 g per group) were first placed in a meat grinder and ground with 1.2 g NaCl and 15 g ice water. Then, different amounts (0%, 3%, 6% and 9%, *w*/*w*) of pea protein were added. All the integrates were transferred to test tubes and dispersed for 3 min at 6500 rpm in a homogenizer under ice bath conditions (T18, IKA, Germany) to prepare minced meat for DMP.

### 2.2. Dynamic Rheological Measurements

The rheological characteristics were measured with a rheometer (MCR301 Converter Company, Anton Paar, Graz, Austria) in oscillatory mode. The rheological parameters were as follows: the starting temperature was 20 °C, the end temperature was 80 °C and the temperature rose linearly at a heating speed of 2 °C/min and the cooling rate was 5 °C/min. The round plate probe of stainless steel was 50 mm, the gap was 1.0 mm, the frequency was 0.1 Hz and the strain was 2%. Each group of minced meat containing 0%, 3%, 6% and 9% was then evenly applied on the lower plate successively. Before the test, a drop of paraffin oil was added to the edge of the plate to separate the sample from the outside air and prevent the sample from evaporating due to heat. The storage modulus (G′) and loss modulus (G″) were then collected for analysis.

### 2.3. Cooking Yield and Water-Holding Capacity

Cooking yield was measured according to Berry et al., (1996) with minor modifications [19]. About 35 g of prepared minced meat for meat batters was then loaded into a 50-mL centrifuge tube, weighed and recorded, then centrifuged at 3000× *g* for 10 min at 4 °C for air removal, and then, the DMB was prepared as follows: heated at 85 °C in a water bath for 30 min, cooled at room temperature after 30 min and, finally, kept at 4 °C for 12 h afterward. After that, the exudation was poured off, the tubes were thoroughly drained and weighed. The cooking yield was calculated according to the ratio of the weight after water loss to the original weight (%) and expressed using the average of three repetitions.

The water-holding capacity (WHC, %) of the meat batters was measured, referring to the method of Kocher and Foegeding (1993), making some modifications [20]. Meat batters containing 0%, 3%, 6% and 9% pea protein, with weight-containing centrifuge tubes, are recorded as m_1_. After being centrifuged for at 4 °C, 5000× *g* for 10 min, then dried, the water on the surface with filter paper was weighed, the weight-containing centrifuge tube was recorded as m_2_. The weight of the centrifuge tube was weighed later as m. The WHC (%) was expressed using the average of three repetitions and calculated according to the formula illustrated as follows:

(1) $$WHC\;\left(\%\right)=\frac{m2-m}{m1-m}\times 100\%$$

### 2.4. Color Measurement

Colors were measured with a CR 400 colorimeter (Minolta Camera Co., Osaka, Japan) and calibrated with a standard plate (L* = 28.97, a* = 0.47, b* = −0.30) before use. The color measurements were carried out in triplicate on a surface of meat batter after determining the cooking yield. The L*, a* and b* values were then recorded.

### 2.5. Texture Profile Analysis (TPA)

The TPA model containing 0%, 3%, 6% and 9% pea protein was determined by texture instrument (TA-XT plus, Stable Micro Systems, Surrey, UK). Parameter settings: the speed range of the pretest was 1 mm/s, the resistance value after the test speed was 5 mm/s, the compression value was 40%, the time range was 5 s, trigger force type was automatic, and the trigger ability was set at 5 g. The indexes of hardness (N), springiness, cohesiveness (ratio), gumminess and chewiness (N) were measured and were expressed using the average of three repetitions.

### 2.6. LF-NMR Relaxation Measurements

Three grams of samples were settled in a test bottle (15 mm diameter × 30 mm height), and the T_2_ relaxation time of the sample was determined using an NMR analyzer (MesoMR23-060H-1, Niumag Electric Co., Shanghai, China), referring to Han et al. (2014) with slight modifications [21]. Experimental parameter settings: CarrePurcelleMeiboomeGill with 16 scans and proton resonance frequency of 22.6 MHz. The NMR data were primarily analyzed by continuous distribution inverse and discrete exponential fittings. The average of three measurements of each sample was recorded

### 2.7. Scanning Electron Microscopy (SEM)

The heat-induced protein gel was cut into small pieces (5 × 5 × 1 mm) and fixed with 2.5% glutaraldehyde for 24 h. Each sample was freeze-dried and sputter-coated with 10 nm of gold. Gels were then analyzed with a Hitachi S-3000N scanning electron microscope (Tokyo, Japan) at an accelerating voltage of 20 kV.

### 2.8. Statistical Analysis

One-way analysis of variance (ANOVA) and Duncan’s multiple range test for statistical analysis and principal component analysis (PCA) were conducted using SPSS^TM^ software (version 20, SPSS Inc., Chicago, IL, USA).

## 3. Results and Discussion

### 3.1. Color

**Figure 1:**
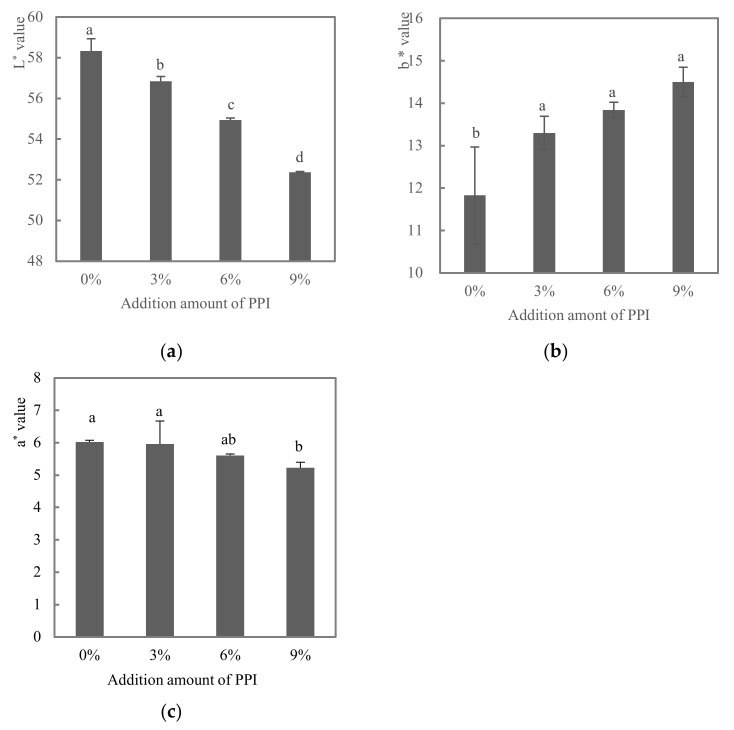
Effect of addition of pea protein on the L* value (**a**), b* value (**b**) and a* value (**c**) of duck meat batters. Note: 0%, 3%, 6% and 9% indicated the levels of addition of PPI to DBM. Data were expressed as the mean ± SD (*n* = 3). Different letters (a–d) indicate a significant difference (*p* < 0.05).

As shown in Figure 1a, with the increase of PPI, a decreasing trend (*p* < 0.05) in L* value can be observed, from 52.4 to 46.4. b* value refers to the yellow value. From Figure 1b, it can be seen that the b* value tends to increase with the increase of pea protein addition. There was a significant difference between the 0% and 3% groups (*p* < 0.05). However, no significant differences were found between 3%, 6% and 9% (*p* > 0.05). The pea protein itself was light yellow in color and the yellow value gradually increased with the increase in the amount of pea protein, which also led to the darkening of the gel. Figure 1c shows that the a* value, which indicates the redness value, decreased with the increasing amount of pea protein added, and the difference between the 0% and 9% groups was significant (*p* < 0.05), with a value decreasing from 6.02 to 5.23. Akesowan (2010) observed a similar trend, where the addition of vegetable protein resulted in a decrease in brightness (L*) and an increase in yellowness [22]. Previous results also showed that the addition of soy flour led to a decrease in the brightness of the burgers [23]. Color is one of the most important bases for making visual judgments about meat products. In conclusion, the color measurements suggest that partial replacement of ground meat with pea protein may lead to a more yellowish color of cooked meat batters. Additionally, the reduction in red color may be due to the addition of non-meat proteins resulting in a lighter, less red product compared to the all-meat control [24].

### 3.2. Cooking Yield and Water-Holding Capacity (WHC)

**Figure 2:**
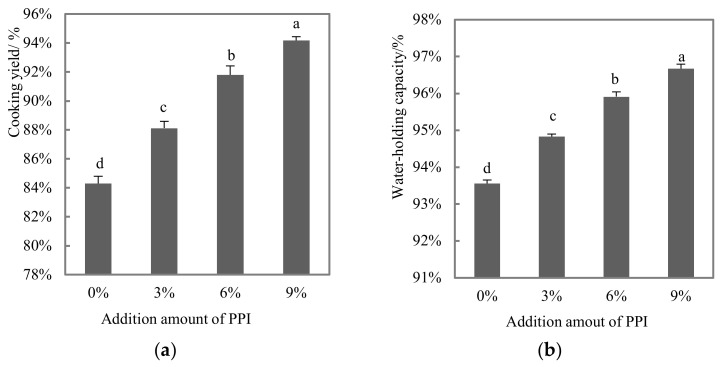
Effect of a pea protein addition level on the cooking yield (**a**) and water-holding capacity (**b**) of duck meat batters. Note: 0%, 3%, 6% and 9% indicated level of addition of PPI to DBM. Data of the cooking yield are the water-holding capacity, expressed as the mean ± SD (*n* = 3). Different letters (a–d) indicate a significant difference (*p* < 0.05).

As seen in Figure 2a, the cooking yield of DMB gradually increased with the increase in the amount of pea protein (*p* < 0.05). The maximum cooking yield (9% group) was 94.17%, which was about 1.1 times higher than that of the control group. It is speculated that the proteins of duck and pea were deformed and aggregated due to enhanced interaction, resulting in a stable and tightly packed mesh structure that binds water molecules and enhances the water-holding capacity, increasing the cooking yield of the gel during heating. WHC was associated with the ability of the system to bind water after protein denaturation and aggregation. From Figure 2b, the WHC of DMB containing pea protein (3–9%) was higher than that of the control group (*p* < 0.05). During heating, pea proteins are highly hygroscopic [25], and the interaction between pea proteins and duck proteins becomes stronger, swelling and restraining the movement of water molecules, resulting in a rigid mesh structure that enhances WHC by retaining water [26]. Pea protein isolates (two main components: Vicilin, 7 s and Legumin, 11 s) can increase the contact area with water, thus improving the water binding capacity of proteins in duck meat, which contains recognizable fleshy fibrous structures [27,28]. Similar findings also showed that higher κ-carrageenan and sodium caseinate contents could also reduce cooking losses in pork muscle gel, thus increasing the water-holding capacity [29]. The results of this study are consistent with previous findings that all non-meat protein treatments resulted in lower cooking losses compared to the all-meat control group [24].

### 3.3. Dynamic Rheological Properties

**Figure 3:**
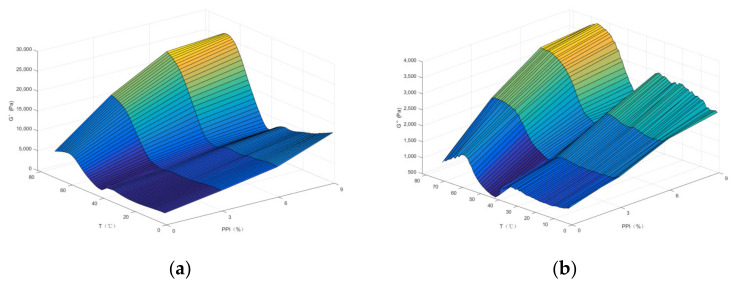

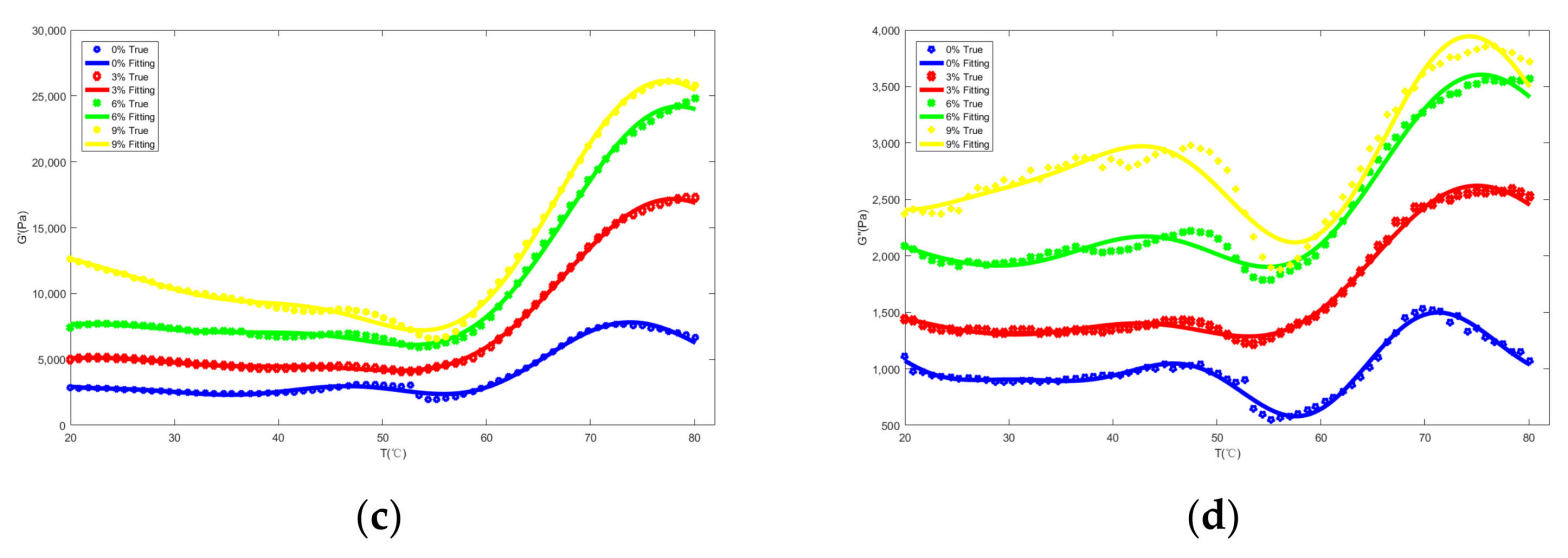
Three-dimensional surface of G′ (**a**), G″ (**b**) with a PPI addition level and temperature of DBM and the Fourier function fitting curves between G′ (**c**) and G″ (**d**) with the temperature of DBM under a different PPI addition level.

According to our results, the occurrence and formation of gels went through two main stages. One was the rapid unfolding of proteins due to heat denaturation, and under the mechanical effect of cross-linking, the proteins formed gels again through the interactions between head dimers; after which, the elastin network structure was slowly formed. The storage modulus G′ can accurately reflect not only the unfolding and cross-linking process of protein molecules at different temperatures but also the elasticity and strength of the protein gels [30]. As can be seen from Figure 3a, the trend of G′ of various minced meats changed approximately the same throughout the heating process after the addition of pea protein. First, G′ decreased slowly between 20 and 41 °C. With the increase of temperature, G′ gradually increased from 41 °C to 46 °C. The first peak could be reached at 46 °C, which should also be the starting point of gel network formation at this moment; the dimer of myosin is mainly a network structure formed by the cross-link between the parts of the head, and when the heating continued, the G′ value of the gel gradually decreased and reached the lowest value around 54 °C, which may be because the tail of the myosin molecule degraded and the protein molecules recombined, thereby destroying the previous protein network structure. Then, when the temperature was averaged between 55 °C and 80 °C, G′ would rise rapidly and then be in a stable state; at this time, the denaturation of the protein gradually increased and further aggregation and cross-linking occurred, forming a more elastic gel mesh structure. For the batter without pea protein, G′ decreased slowly between 20 and 37 °C and rose between 37 and 48 °C, reaching the first peak at 48 °C; continuing to heat, the G′ decreased and reached a minimum around 55 °C; after which, G′ rose rapidly between 56 and 80 °C. It was evident that the initial and final values of G′ of meat batters containing pea protein (3–9% group) were significantly higher than those of the control group (0% group), and the increase of pea protein improved the ability of duck meat batters to form better gels. The loss modulus (G″) reflects the tendency of the sample to be sticky, which means that the energy loss of the sample at a certain strain is transferred to other forms of energy. Our results showed that the experimental group (3–9%) had a higher viscosity at the beginning and at the end compared to the control group (0%) and increased with the addition of pea protein (Figure 3b). The results of previous studies also showed that the G′ values of dietary fiber added to bamboo shoots were higher than those of the blank control group. The G′ values of pork myofibrillar protein gels increased with the addition of dietary fiber from bamboo shoots [31], which is consistent with the results of this experiment. Dynamic rheological tests at sweeping temperatures (20–80 °C, 2 °C/min) showed that the G′ (modulus of elasticity) of pork myofibrillar protein pre-gel samples also increased substantially with the addition of emulsion [32].

MATLAB (Mathsworks 2016, Natick, MA, USA) was used to fit the G′, G″ versus temperature for DMB with different pea protein concentrations, in accordance with the Ffourier function (Figure 3c,d). It could be seen that the fourier function was used to fit the relationship between G′, G″ and temperature for DMB at different pea protein concentrations with very high fitting accuracy and R^2^ above 0.99 (data not shown). The model can well describe the storage modulus G′ and G″ of DBM versus temperature at different PPI addition levels, which is not a simple linear relationship but a fourier series relationship.

### 3.4. Texture Profile Analysis

As can be seen from Table 1, the hardness, gumminess and chewiness of the DMB showed a continuous increase with the increase of pea protein content. Among all the groups, the maximum hardness (64.53 N), chewiness (51.80 N) and gumminess (49.92) were observed in the 9% PPI addition groups. The differences in hardness, gumminess and chewiness of DMB among groups were significant (*p* < 0.05). However, it should be mentioned that these parameters were similar between 3% and 6% pea protein contents (*p* > 0.05). Cohesiveness and springiness were not affected by the amount of PPI added. Since pea protein has the ability of gel formation and emulsification; thus, the increase of the pea protein addition not only leads to the rise of the number of gel molecules per unit volume but also the probability of intermolecular collision increases significantly, which can promote the gel formation ability, form a stable and elastic network organization and bind more water to improve the quality characteristics of the gel [33]. However, too much pea protein can affect the interaction of pea protein with protein molecules in duck meat; thus, fewer water molecules will be bound, so the hardness will increase and the springiness will decrease. As a result, the springiness and cohesiveness of the gel will first increase and then decrease. The results of previous studies have shown that peanut protein does significantly improve the structural properties of chicken salt-soluble protein gels [34]; the effect of soy protein on the gel properties of beef also showed that its springiness roughly rises first and then decreases as the amount of soy protein added increases, which is consistent with the experimental results presented here [6]. Due to the strong functionality of pea protein, it partially promotes the cross-linking of protein molecules, which contributes to the formation of a better network structure; binds the migration of free water; improves the hardness and recovery value of batters and forms an intermolecular cross-linked network structure during thermal denaturation and solidification. At the same time, it is filled with a large amount of water molecules, showing relatively better elasticity. Previous studies have also reported that increasing the addition of non-meat proteins increases the hardness and viscosity of emulsified meat batters [24], leading to a denser protein matrix and, thus, a stiffer structure. Bloukas and Paneras (1993) reported that hardness values were positively correlated with the protein content of low-fat (10%) frankfurters [35]. Lamb plasma proteins were reported to enhance the gel hardness and improve the sensory quality of emulsified lamb sausages [36]. Similar results have been reported for the myofibrillar protein gel strength of normal and even PSE pork meat when the soy protein levels were increased [37]. Additionally, the addition of carbohydrates such as κ-carrageenan at levels below 2% significantly affected the cooking yield, firmness, adhesion, chewiness, gelling and viscosity of the product [38]. Park et al. [39] also reported that konjac flour was added to surimi (ground fish) to make surimi-based products more heat resistant and consistent in shear stress changes during repeated freezing/thawing cycles.

### 3.5. NMR Proton RELAXATION

**Figure 4:**
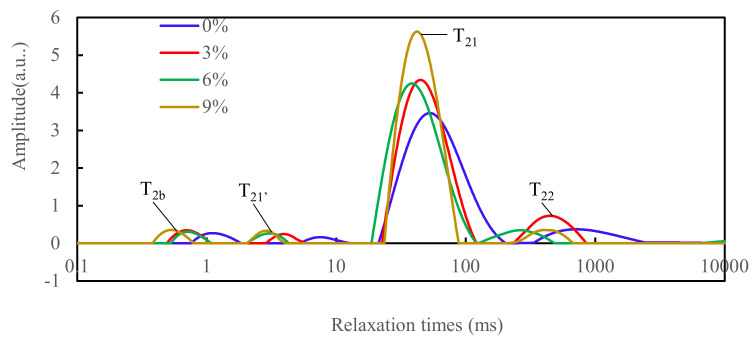
Effect of the pea protein addition levels (0%, 3%, 6% and 9%) on the distribution of the spin–spin relaxation times (T_2_) of duck meat batters.

NMR is an effective method to provide accurate information on the dispersion and transfer of water in a gel system without destroying the gel structure. The T_2_ distribution of DMB with PPI is shown in Figure 4, with four peaks: T_2b_, T_21′_, T_21_ and T_22_ at 0.1–1 ms (commonly referred to as T_2b_; bound water); a minor group between 1 and 10 ms (widely referred to as T_21′_; partially immobilized water); a major group between 10 and 100 ms (denoting T_21_; moderately immobilized water) and after the 100 ms represents free water T_22_. In short, the shorter the time of T_2_, the tighter the water is bound by the protein molecules [40]. As can be seen in Table 2, there was no significant difference between T_2b_ and T_21′_ in all the groups (*p* > 0.05); however, there was a subtle decreasing trend with increasing pea protein addition (0–6%). Table 2 also shows that there was no significant difference in PT_2b_ and PT_21′_ between all the groups, as the amount of pea protein added increased (*p* > 0.05). However, in the experimental group (3–9%), there was an increasing trend in PT_21_ (*p* < 0.05) and a decreasing trend in PT_22_ (*p* < 0.05). In conclusion, the addition of pea protein helped to bind free water well to the reticulum, reducing the proportion of free water and thus increasing the proportion of immobilized water, which, in turn, reduced the water loss during processing and improved the water-holding capacity.

Similar results were reported in our previous study that the water-holding capacity of duck myofibrillar protein gels could be enhanced by the addition of PPI (0.5–2%), since the free water content is inversely proportional to the water-holding capacity [10,41]. Initially, T_2b_, T_21′_ and T_21_ relaxation times were significantly reduced; PT_21′_ and PT_21_ peak ratios were increased and PT_22_ was significantly reduced, respectively, which implied an increase in the immobilized water content. Previous studies have also shown that the use of soybean isolate can produce reduced-salt frankfurters with desirable quality [42], since the water content is closely related to the protein and gel structure. The shorter the relaxation time of water in the different parts, the more tightly it is bound to the protein molecules and the less mobile the water [43].

### 3.6. Microstructure of the Gels

**Figure 5:**
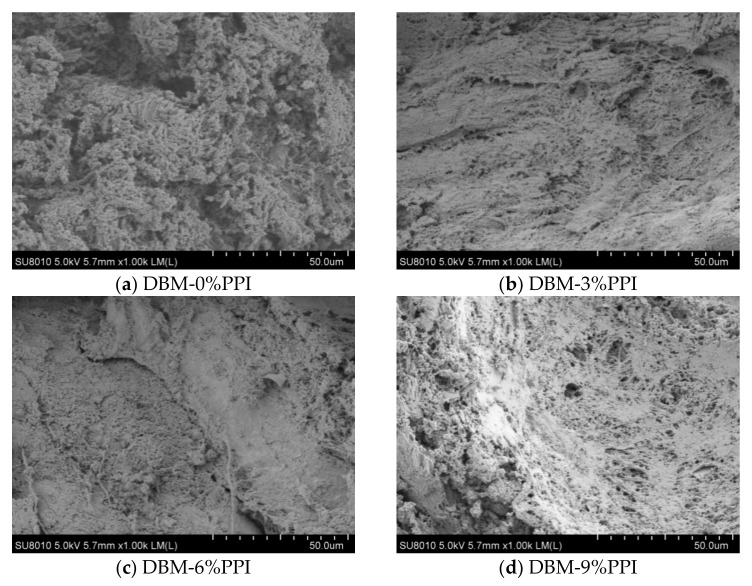
Effect of the pea protein addition level (0%, 3%, 6% and 9%) on the microstructure (Scanning electron microscope, 2000×) of DBM-PPI.

Micrographs of the batters containing PPI (0%, 3%, 6% or 9%) are shown in Figure 5. The results show that the microstructure of the batters became more denser and firmer with the increase of pea protein. Apparently, the control still had voids and pores in the structure, showing a lower density of the protein matrix, and small molecules of components of pea protein would be filled into the above structure. The surface of the protein molecules themselves contain hydrophilic amino acids, which help to retain moisture and stabilize the gel structure. These results can be corresponded to the previous textured structures. Although, the higher the amount of added material, the better the network. However, it is worth mentioning that excessive additions can lead to weakening of the myofibrillar protein pattern structure molecules due to spatial blocking effects. Whether the lack of interaction between pea proteins and meat proteins may be the main obstacle in producing strong gels is unclear. It is hypothesized that pea protein promotes the formation of salt-soluble protein gel structures mainly through physical filling compared to the control. The gel structure with pea protein was significantly improved, and the best gel network structure was formed, which was compact, smooth and largely free of pores, allowing for better fat preservation and water retention. Previous studies have also shown that the size and shape of protein molecules have a crucial influence on the microstructure of protein gels. More aggregated and less homogeneous protein matrix structures were observed in meat emulsions with higher protein contents [44]. Some pea protein molecules formed their regular spherical aggregates that filled the cavity structure of the gel network. Chen et al. suggested that a homogeneous and delicate network structure could better bind water into the gel system and enhance its water retention and other gel properties [45]. Ullah et al. reported that moderate amounts of natto protein could improve the chicken muscle fiber protein gel microstructure and also reduced the cooking loss [46]. Ji et al., also reported that konjac glucans helped Alaskan pollock minced fish to form more compact and homogeneous mixed gels [47] that combined to promote interactions between proteins, water and other molecules to produce more cross-linking into proteins with larger molecular weights and, finally, a rigid three-dimensional network microstructure.

### 3.7. Principal Component Analysis

**Figure 6:**
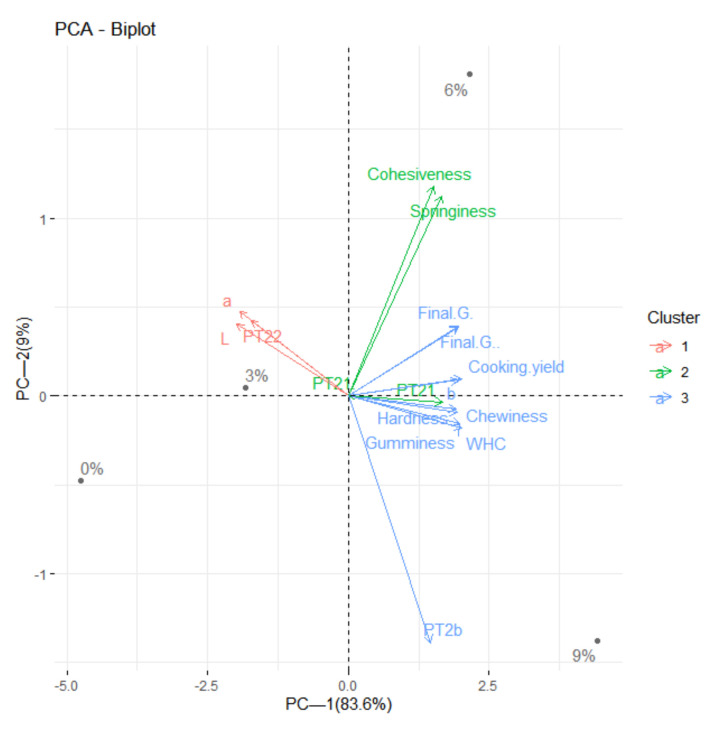
Loading plots and score plots of the groups of the principal component analysis at various levels of the addition of PPI (0%, 3%, 6% and 9%).

The results show that the two principal components explained 83.6% and 9% of the overall variance in the data, respectively (Figure 6). Thus, the model explains 92.6% of the total variance in the data, which shows a strong correlation in the original data. As shown in Figure 6, the first principal component (83.6% of the variance) was mainly positively correlated with PT_21_, WHC, cooking yield, hardness, chewiness and the final G′ and negatively correlated with the L* value, a* value and PT_22_, mainly related to the internal water status, distribution and gel formation ability of the gel. These results suggest that there is an interconnection between water fluidity and texture, which are key factors affecting the gel properties of batters. This could be due to the addition of pea protein isolate, which enhanced the rigid gels with better water-binding ability and gel strength. The second principal component (9% variation) was mainly characterized by two variants (cohesiveness and springiness), which were mainly related to the texture of the batters. Finally, the results clearly indicate that the water distribution is closely related to cohesiveness [48]. A cluster analysis clearly showed that there were differences among different groups (0–9%). Previous studies have also shown that the increase in non-meat protein content facilitates the formation of a stable elastic network structure. In conclusion, the addition of pea protein can well bind free water into the network structure and reduce the proportion of free water, thus increasing the proportion of water that does not easily migrate, improving the water-holding capacity of the batters and, thus, forming a rigid three-dimensional gel structure.

## 4. Conclusions

The addition of pea protein improves the forming ability of duck breast mince and significantly enhances the properties of duck meat batters, increasing the water retention, cooking yield, rheological properties and textural properties of the matrix. As the amount of pea protein increases, the gel hardness, gumminess and chewiness increase, and springiness and cohesiveness first rise and then fall, facilitating the formation of a stable elastic network structure. Further studies are needed to determine the effect of protein–protein interactions on gel properties to investigate the mechanisms and provide more opportunities to expand the utilization of pea protein in duck products.

## Figures and Tables

**Table 1:** Effect of the pea protein addition level (0%, 3%, 6% and 9%) on the hardness, springiness, cohesiveness, gumminess and chewiness of the duck meat batters.

Addition Level	Hardness (N)	Springiness	Cohesiveness (Ratio)	Gumminess	Chewiness (N)
0%	25.56 ^c^ ± 8.07	0.907 ^a^ ± 0.003	0.791 ^a^ ± 0.067	21.94 ^c^ ± 7.22	23.20 ^c^ ± 6.54
3%	49.12 ^b^ ± 1.63	0.916 ^a^ ± 0.064	0.792 ^a^ ± 0.004	35.30 ^b^ ± 0.86	38.98 ^b^ ± 1.05
6%	53.29 ^b^ ± 2.67	0.960 ^a^ ± 0.008	0.811 ^a^ ± 0.004	40.90 ^b^ ± 2.03	43.35 ^b^ ± 2.35
9%	64.53 ^a^ ± 3.32	0.938 ^a^ ± 0.003	0.802 ^a^ ± 0.018	49.92 ^a^ ± 1.56	51.80 ^a^ ± 1.36

Note: 0%, 3%, 6% and 9% indicate a level of addition of PPI to DBM. Data were expressed as the mean ± SD (*n* = 3). Different letters (a–d) in a row indicate a significant difference (*p* < 0.05).

**Table 2:** The relaxation times and corresponding peak areas of duck meat batters with the addition of the pea protein (0%, 3%, 6% and 9%).

	0%	3%	6%	9%
T_2b_ (ms)	0.96 ^a^ ± 0.26	0.91 ^a^ ± 0.35	0.76 ^a^ ± 0.09	0.67 ^a^ ± 0.29
PT_2b_ (%)	2.58 ^a^ ± 0.82	3.03 ^a^ ± 0.61	2.96 ^a^ ± 0.49	3.55 ^a^ ± 0.60
T_21′_ (ms)	4.76 ^a^ ± 2.45	4.84 ^a^ ± 2.26	3.33 ^a^ ± 0.24	2.83 ^a^ ± 0.64
PT_21′_ (%)	2.03 ^a^ ± 0.71	1.96 ^a^ ± 0.68	2.31 ^a^ ± 0.51	2.11 ^a^ ± 0.11
T_21_ (ms)	50.98 ^a^ ± 1.50	45.13 ^b^ ± 0.87	38.69 ^d^ ± 0.01	41.79 ^c^ ± 0.00
PT_21_ (%)	85.64 ^c^ ± 0.52	83.57 ^d^ ± 0.38	88.62 ^b^ ± 0.12	89.68 ^a^ ± 0.11
T_22_ (ms)	687.52 ^a^ ± 33.09	447.25 ^b^ ± 4.98	264.47 ^d^ ± 0.00	393.51 ^c^ ± 8.79
PT_22_ (%)	8.86 ^b^ ± 0.94	11.43 ^a^ ± 1.29	5.87 ^c^ ± 0.40	3.32 ^d^ ± 1.22

Note: Data were expressed as the mean ± SD (*n* = 3). Different letters in row indicate significant differences (*p* < 0.05).

## Data Availability

The datasets generated for this study are available upon request from the corresponding author.
